# CRASA: Chili Pepper Disease Diagnosis via Image Reconstruction Using Background Removal and Generative Adversarial Serial Autoencoder

**DOI:** 10.3390/s24216892

**Published:** 2024-10-27

**Authors:** Jongwook Si, Sungyoung Kim

**Affiliations:** 1 Department of Computer AI Convergence Engineering, Kumoh National Institute of Technology, Gumi 39177, Republic of Korea; jwsi425@kumoh.ac.kr; 2 Department of Computer Engineering, Kumoh National Institute of Technology, Gumi 39177, Republic of Korea

**Keywords:** GAN, autoencoder, image reconstruction, disease diagnosis, anomaly detection, GrabCut, smart farm

## Abstract

With the recent development of smart farms, researchers are very interested in such fields. In particular, the field of disease diagnosis is the most important factor. Disease diagnosis belongs to the field of anomaly detection and aims to distinguish whether plants or fruits are normal or abnormal. The problem can be solved by binary or multi-classification based on a Convolutional Neural Network (CNN), but it can also be solved by image reconstruction. However, due to the limitation of the performance of image generation, SOTA’s methods propose a score calculation method using a latent vector error. In this paper, we propose a network that focuses on chili peppers and proceeds with background removal through GrabCut. It shows a high performance through an image-based score calculation method. Due to the difficulty of reconstructing the input image, the difference between the input and output images is large. However, the serial autoencoder proposed in this paper uses the difference between the two fake images, instead of the actual input, as a score. We propose a method of generating meaningful images using the GAN structure and classifying three results simultaneously by one discriminator. The proposed method showed a higher performance than previous research, and image-based scores showed the best performance.

## 1. Introduction

A smart farm refers to an intelligent farm that can automatically manage the growing environment of crops and livestock by applying convergent technologies such as unmanned automation, artificial intelligence, and big data to greenhouses and livestock. It is a technology that can adapt to any situation based on assessing the environment or the biometric information of the production facilities. It can maximize productivity through minimal labor and energy. These smart farms contribute to production and distribution using environmental, crop, and soil sensors. In the rural areas of countries experiencing aging societies, there is a risk to the sustainability of related industries due to the aging of the population engaged in the agricultural and livestock sectors, coupled with a decrease in the inflow of young people. The overall production population is decreasing due to the decline in the fertility rate, which has emerged as a social problem, and, in particular, the elderly population of farmers continuously increasing yearly. Therefore, smart farms are recognized as a key means to strengthen agricultural competitiveness and secure various age groups of farmers through an inflow of youth. In particular, artificial intelligence solves many agricultural challenges and uses technologies such as machine learning, computer vision, and predictive analysis. The most productive aspect of smart farms is crops. To manage crops in a smart farm environment, it is necessary to continuously create an environment that is most appropriate for the crop, and the diseased crops should be excluded from production. In this paper, we want to pay attention to chili peppers among various crops, and the purpose of this paper is to check the condition of the crops using computer vision technology to classify whether they are normal or diseased.

There are two main methods of determining the state of crops using computer vision technology. The most frequently used method is determining the input image after converting the result for the last layer through several convolution layers to a value between 0 and 1 through an activation function. Furthermore, based on the autoencoder structure, image reconstruction is performed with an image that follows a normal data distribution using only normal data, and the state of the crop can be determined using errors in the input/output results. However, the image reconstruction method is relatively low in accuracy because it is difficult to reconstruct accurately. Attempts have been made to apply autoencoders to Generative Adversarial Networks (GANs) [1] to overcome these shortcomings, yet significant performance improvements have not been achieved [2,3]. However, such efforts have laid the groundwork for further research to enhance performance through architectural modifications or by proposing new processes [4,5,6,7,8,9,10,11,12,13]. Due to the limitation in accuracy, although performance improvement has been achieved more than before, recent researchers have made significant performance improvements using latent vectors between the encoder and decoder. In this paper, we present a novel framework that overcomes the limitations of performance improvement in the image domain rather than the latent vector and has a higher accuracy. We define the proposed method, “**C**hili Pepper Disease Diagnosis via Image Reconstruction Using Background **R**emoval and Generative **A**dversarial **S**erial **A**utoencoder”, as **CRASA**.

The contribution of this paper is as follows.

- We present a new GAN-based framework for detecting crop anomalies using the errors between image domains in image reconstructions.
- By connecting two autoencoders in series, a new method of diagnosing normal and diseased crops using two reconstructed images is presented, and the performance is higher than that of previous research.
- To reduce the result of misclassification, the background of the chili pepper image may be removed through a GrabCut algorithm in the image processing field.

In Section 2, previous studies related to anomaly detection based on image reconstruction and the diagnosis of plant diseases are introduced, and the differences between these approaches and the proposed method are analyzed. Section 3 provides a detailed explanation of the proposed method, including its structure and specific diagnostic approach. In Section 4, the performance of the proposed method is analyzed and validated through a comparison with previous research. Finally, Section 5 presents the conclusion and suggests future research directions, discussing insights into the proposed method and potential areas for further investigation.

## 2. Related Works

After a GAN [1] was first presented, many researchers began to develop and apply it. This structure, in which the generator and the discriminator are opposed to each other to improve the performance, was mainly used in the image generation field. Although anomaly detection was still common for classifications via the image processing field or Convolutional Neural Networks (CNNs), researchers began to apply GANs to anomaly detection, deviating from existing ideas.

### 2.1. Anomaly Detection via Image Reconstruction

AnoGAN [2] was the first unsupervised learning method to detect outliers using only normal image data. With a GAN-based structure, this study added residual loss to allow generators to train manifolds of normal data and to reduce the difference between inputs and outputs. Discrimination loss includes a feature-matching process that identifies the probability distribution of the input data, identifies real and fake data, and proceeds with learning to follow the normal data distribution. It was noted as the first idea based on GANs for anomaly detection, and many GAN-based anomaly detection research studies have been actively conducted. By improving this AnoGAN [2], the two-step method of f-AnoGAN [2], in which encoder training is added after GAN learning, emerged. f-AnoGAN [3] conducts encoder training for the latent space mapping of data to obtain the result of the feature extraction and generates the result of making it the input of the generator again to improve speed and performance. To overcome the shortcomings of the autoencoder, which makes it difficult to find satisfactory results due to the reconstruction of abnormal areas, the problem is solved by searching for and generating the most relevant items based on the memory of normal data using memAE [3]. SALAD [5] proposed a novel anomaly detection framework with image reconstruction that considers both image and latent spaces. Only useful information can be reconstructed from normal data by adding a loss function using SSIM and a loss function that constrains the center. SSM [6] is a framework that adds random masking and restoration to the autoencoder structure, which proposes a method to improve the image reconstruction results. This research enhances the learning of inpainting and can locate abnormal areas through masks of various sizes. J. Si et al. [7] proposed a method of determining defects through an image reconstruction method of a thermal image of a solar cell. This research was evaluated using only some pixels of the subtraction image that best represented the characteristics and showed a higher performance than the patch method. J. Liu et al. [8] presented a GAN-based network for fiber defect detection. The proposed network is trained in multiple stages and has been confirmed to have a high defect detection performance under various conditions. V-DAFT [9] applies the Fourier transform with a normal reconstructed template to the resulting image to solve the problem that the image reconstruction results of the denoising autoencoder structure differ significantly from the input. The applied results predominantly retain features with substantial differences, enhancing the anomaly detection performance. DAGAN [10] proposed research in anomaly detection that solves the problem of an imbalance between samples. DAGAN [10] introduced an autoencoder structure to the generator and the discriminator, improving the stability of learning and the performance of image reconstruction.

In the field of anomaly detection, notable reconstruction-based studies include DFR [11], DRAEM [12], and DDAD [13]. DFR [11] focuses on detecting anomalies in very small regions, proposing a method that combines multiple feature maps capable of representing various spatial information for reconstruction. This approach allows for effective anomaly detection by reconstructing multiple feature maps rather than the image itself. DRAEM [12] deviates from reconstructing normal images and instead generates anomalous images, which are then reconstructed into normal images. This process helps to address data imbalance issues in anomaly detection, enabling a higher performance. DDAD [13] is a study that combines diffusion modeling with anomaly detection, allowing it to operate in a conditional format. This model generates images based on diffusion processes and extracts features, achieving a high performance in detecting anomalies even in minute areas. However, since DFR [11], DRAEM [12], and DDAD [13] have been evaluated specifically in the manufacturing sector, their application across various fields with a high accuracy is challenging. Yet, if trained appropriately for specific data domains, they can yield excellent results.

### 2.2. Diseased Plant Diagnosis

Research in disease diagnosis can be conducted using GAN-based methods, but due to challenges in achieving a high performance, most studies predominantly employ CNN-based classifiers or object detection frameworks. DoubleGAN [14] proposed a GAN structure to detect diseases in plants. This research consists of two steps. Step 1 uses both normal and diseased plants, and, based on WGAN [15], data augmentation is carried out using a pretrained model. Subsequently, in Step 2, the number of diseased leaves data is expanded by increasing the image size by 16 times using SRGAN [16]. It uses several extended GANs to improve the performance. Punam Bedi et al. [17] proposed a hybrid model for detecting plant diseases by combining a Convolutional AutoEncoder (CAE) and a CNN. This creates a network that can reconstruct images through the CAE structure and simultaneously focuses on the compressed domain representation, the output of the encoder. This domain is a latent vector used by the CNN to classify normal or diseased plants. This study achieved a high performance with the training of only 9914 parameters. Sharath D. M. et al. [18] proposed a framework for detecting plant diseases using data preprocessing and a CNN for the image processing. They used the GrabCut segmentation method proposed by Yubing Li et al. [19] for background removal, which improves upon the GrabCut [20] technique.

Furthermore, morphology errors are used to eliminate noise. These preprocessed data are provided to the user through a mobile application with nine labels through a CNN classifier. A. Abbas et al. [21] introduced how to generate synthesized images using a C-GAN [22] to detect diseases in tomatoes and to differentiate 10 diseases through transfer learning in DenseNet121 [23]. This method showed a very high classification accuracy and the effect of data augmentation on classification performance improvements. R. Katafuchi et al. [24] proposed a method for detecting plant diseases based on the ability to restore colors. We used Pix2pix [25] to print the results of the original restoration and present a new anomaly score based on CIEDE2000 [26] to improve the performance. T. Tosawadi et al. [27] proposed a method of detecting disease in rice plants. This research can improve the performance of disease recognition in small areas by selecting and classifying multiple sections from diseased areas.

## 3. Proposed Method

The proposed method primarily aims to determine the presence or absence of disease by overcoming the limitations of previous reconstruction techniques to enhance performance. This is achieved by overcoming the constraints of GAN-based reconstruction capabilities through the serial arranging generators.

It is evaluated by comparing the results of generators and converting these comparisons into scores for assessment. For disease diagnosis, we propose a scoring method using the errors between images. The preprocessing step incorporates the application of GrabCut [20] to enhance the clarity and effectiveness of disease diagnosis in chili peppers. This technique excels at isolating the subject from the background in images taken in real farm environments, significantly reducing the potential for misclassification errors.

### 3.1. Preprocessing

**Figure 1:**
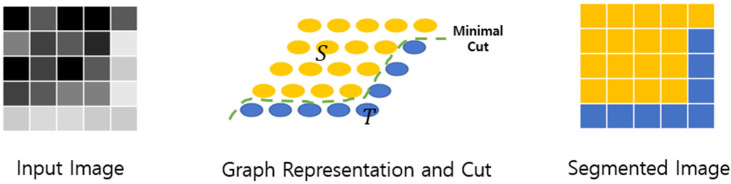
GrabCut process.

The background of an input image is removed to reconstruct only useful information by leaving only chili peppers in the image. The technology used is GrabCut [20]. GrabCut [20] is an algorithm based on the division of regions through the minimal cut algorithm used in the graph algorithm. Segmentation is possible by assuming the pixel of the image as the vertex of the graph and dividing it into foreground and background groups to find the optimal cut. The GrabCut process is shown in Figure 1.

In a precise context, GrabCut [20] is an algorithm designed for foreground object extraction, offering the capability for user interaction to refine the output. Nevertheless, given the need for automated disease diagnosis in this study, the technique is adapted to automatically isolate areas containing chili peppers without user intervention. Despite this automation, GrabCut does not guarantee flawless results; hence, supplementary efforts involving histogram-based analysis are implemented to enhance the accuracy of foreground segmentation. When the foreground is not distinctly represented in the initial image, histogram equalization is applied across the entire image to modify the pixel distribution, thereby improving the visibility of foreground elements.

**Figure 2:**
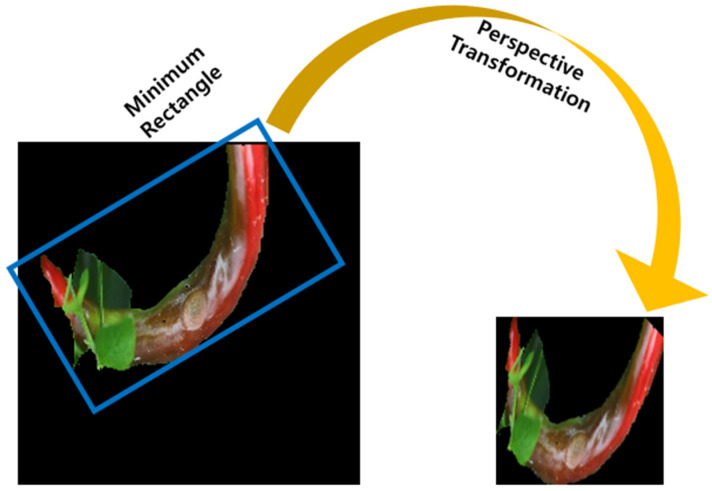
The process of perspective transformation by cropping only the chili pepper area.

The input of the deep learning network is the image of the chili pepper region from which the background has been removed. Preprocessing is required to distribute the chili pepper regions in the best state. After removing noise and filling holes in the image through mathematical morphology, the minimum bounding box rotated by the angle to include the minor background area is found. An input image with a size of 128 × 128 is generated through a perspective transformation in this rectangle area, and the process is shown in Figure 2.

### 3.2. Detailed Process of the Proposed Method

**Figure 3:**
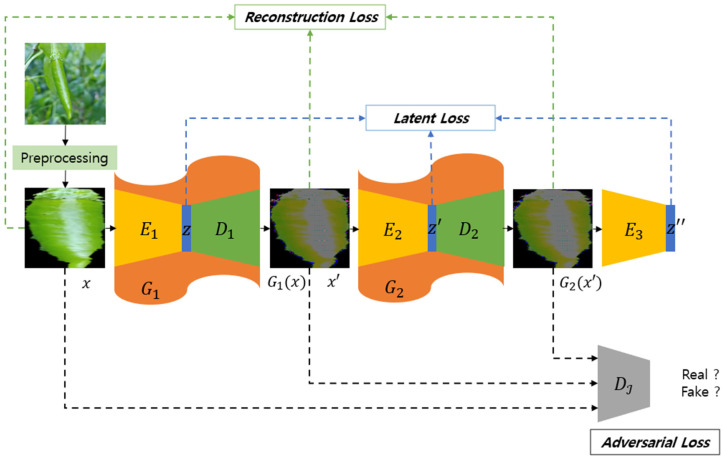
Overall training architecture.

The methodology introduced in this paper uses Ganomaly [28] as a baseline. It achieves enhanced outcomes by addressing the limitations encountered in prior research on anomaly detection via image reconstruction, specifically using an autoencoder architecture. This approach involves compressing an input image into a latent vector and reconstructing it. The underlying hypothesis is that, while the network is trained to compress and reconstruct normal images efficiently, a significant disparity will be observed in the reconstruction of anomalous images. Nevertheless, due to the inherent constraints of the network, despite preserving essential features and low-level content, minimizing the discrepancy between the input and reconstructed images remains challenging. To mitigate this shortcoming, this paper presents a novel architecture that sequentially integrates two autoencoders (Figure 3), demonstrating a strategic improvement over traditional single autoencoder models.

The training process for the network outlined in this paper is exclusively conducted using images of healthy chili peppers. In contrast, healthy and diseased chili pepper images are employed during testing. The generator within the network is tasked with creating two synthetic images that mimic the appearance of the actual input images closely enough to deceive the discriminator. The discriminator, in turn, can differentiate between the input image and the two images produced by the generator. As a result, the image reconstruction approach outlined herein demonstrates a superior performance relative to previous studies that relied on anomaly detection through latent vector-based scoring methodologies. This method signifies that, to surpass the performance limitations of reconstruction, it enables achieving a higher performance through comparisons between images generated by the generator, rather than the traditional approach of comparing with the original images.

The training dataset $D_{trn}$ consists of only N normal chili pepper images in large quantities. The test dataset $D_{tst}$ consists of $M_{1}$ normal chili peppers and $M_{2}$ diseased chili peppers. The labels on the normal datasets are all labeled zero because only normal images exist (i.e., $D_{trn}=\{\left(x_{1},y_{1}\right),\ldots ,\;(x_{N},y_{N})\}$), where y∈ {0}). The test datasets are labeled zero for normal (${x_{N}}_{i}$) and one for diseased (${x_{D}}_{i}$) chili pepper images (i.e., $D_{tst}=\{\left(x_{N_{1}},y_{N_{1}}\right),\ldots ,\;(x_{N_{M_{1}}},y_{N_{M_{1}}}),\left(x_{D_{1}},y_{D_{1}}\right),\ldots ,\;(x_{D_{M_{2}}},y_{D_{M_{2}}})\}$), where y∈ {0, 1}). Here, x represents the data sample, and y denotes the ground truth label. For the training, we modified this to follow a standard normal distribution to approximate all the data, such as X~N(0, 1), and we made the test data follow a normal distribution based on the mean and variance of the training datasets, such as $D_{tst}\sim N(X_{tst}-\bar{X},\;\sigma_{X})$.

Since the generator ($G_{1},G_{2})$ is composed of an autoencoder structure, it can be separated into an encoder and a decoder. The encoder ($E_{1},\;E_{2},E_{3}$) compresses the input image to generate a P-dimensional latent vector that can be well represented, and the decoder ($D_{1},\;D_{2}$) uses this latent vector ($z,\;z^{\prime },\;z^{\prime \prime })$ as an input to restore it to the result closest to the input image of the encoder. $z,\;z^{\prime }$, and $z^{\prime \prime }$ mean the latent vector that the encoder best expresses $x,\;x^{\prime }$, and $x^{\prime \prime }$, and the difference between the latent vectors should also be reduced because the input image is compressed. Accordingly, it has the same correlation as $z=E_{1}\left(x\right)$, ${z^{\prime }=E}_{2}\left(x^{\prime }\right)=E_{2}\left(G_{1}\left(x\right)\right)$, and ${\;z}^{\prime \prime }=E_{3}\left(x^{\prime \prime }\right)=E_{3}(G_{2}\left(x^{\prime }\right))$.

The first generator is defined as $G_{1}$, and the second generator is defined as $G_{2}$. $G_{1}$ aims to produce real input images similar to the limits that the network can generate, and $G_{2}$ trains to produce the same results as $G_{1}$. Accordingly, it has the same correlation, such as $x^{\prime }=G_{1}\left(x\right)=D_{1}(E_{1}\left(x\right))$ and ${x^{\prime \prime }=G}_{2}\left(x^{\prime }\right)=G_{2}\left(G_{1}\left(x\right)\right)=D_{2}(E_{2}\left(G_{1}\left(x\right)\right)$. It is possible to minimize the errors between images by connecting the two series generators, $G_{1}$ and $G_{2}$, of this structure. The first reconstructed image $G_{1}(x)$ has a huge difference from the input image and a small difference in error with the abnormal image. However, this result is eventually generated by the output of the network and produces an image as close as possible, although not identical, to the input image x. Therefore, generating one more time based on the output image of the first network $G_{1}$ can show a tiny error and a more definite difference. The discriminator $D_{I}$ determines the real input image and the two results generated by the generator $G_{1}$ and $G_{2}$. From the standpoint of the discriminator $D_{I}$, the real image should be determined as a fake image and the image generated by the generator $G_{1}$ and $G_{2}$. However, the generator must produce more realistic results to distinguish the discriminator from the other way around to deceive it.

The generator ($G_{1}$, $G_{2}$) and discriminator ($D_{I}$) models are designed based on the encoder–decoder architecture proposed in Ganomaly [28], serving as a baseline for the network structure. Table 1 illustrates the proposed model architecture in this study. The model features a deep structure with repeated iterations of identical modules. Notably, the three encoders and one discriminator share the same architectural design. The input and output layer sizes are set to (128, 128, 3). They are configured similar to (128, 128, 3), maintaining the same dimensions as the input through the encoder–decoder architecture. All convolutional operations employ 4 × 4 kernels, utilizing the ‘same’ padding across all layers, except for the latent vector. The latent vector dimension is (1, 1, 100), for which the ‘valid’ padding is employed to adjust the size. In the encoder and discriminator, all activation functions are implemented using Leaky-ReLU. In contrast, Tanh is used only in the decoder in the final layer, while ReLU is employed in the remaining layers.

**Figure 4:**
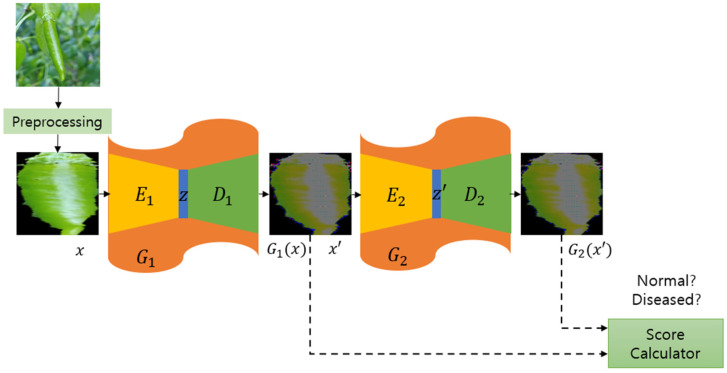
Test architecture using score calculator.

After sufficient training has progressed, determining whether the input image is normal or diseased is required. In the same way as the training framework, after outputting all results, the abnormal score is calculated using only two images generated by the generator, shown in Figure 4.

The score calculator computes the L_2_ loss for each RGB image, where $G_{1}\left(x\right)$ and $G_{2}(x^{\prime })$ are three-dimensional color images produced by the generator. When the error e for each image is calculated, it is converted into a final score through a normalization process, as shown in Equation (1). This score means that the closer to zero, the smaller the error and the normal pepper image. Depending on the distribution of the entire experimental data, the point at which the True Positive Rate (TPR) and False Positive Rate (FPR) are maximized is designated as a threshold value τ in the test stage to finally determine whether the chili pepper image is normal or diseased, as shown in Equation (2).

(1) $$S\left(G_{1}\left(x\right),G_{2}\left(x^{\prime }\right)\right)=\frac{e-min(e)}{\max\left(e\right)-min(e)}$$

(2) $$\left\{\begin{array}{c} if\;\;\;\;\;\;\;\;\;\;S\left(G_{1}\left(x\right),G_{2}\left(x^{\prime }\right)\right)<\tau \;\;\;\;\;\;\;\;:\;x=“\mathrm{Normal}”\\ \;\;\;else\;\;\;\;\;\;\;\;\;\;\;\;\;\;\;\;\;\;\;\;\;\;\;\;\;\;\;\;\;\;\;\;\;\;\;\;\;\;\;\;\;\;\;\;\;\;\;\;\;\;\;\;\;\;:\;x=“\mathrm{Diseased}”\;\; \end{array}\right.$$

The loss function proposed in this paper is composed mainly of three losses. Adversarial loss $L_{adv}$ is a loss to the overall training of the framework based on the GAN structure. The generator and the discriminator are at odds with each other and serve to lead to better results. The discriminator has the performance of simultaneously classifying two real and one fake image, with the fake output close to zero and the real output close to one. Binary cross-entropy, commonly used for binary classifications, is applied to each result and is represented by Equation (3). Only the discriminator applies a loss function such as Equation (4), where Equation (3) is used, and the generator defines the loss function, so that the difference in each result is minimized based on the L2 loss. It is represented by Equation (5).

(3) $$L_{BCE}\left(a,b\right)=-\frac{1}{N}\sum_{i=0}^{N-1}\left[b^{\left(i\right)}loga^{\left(i\right)}+\left(1-b^{\left(i\right)}\right)\log\left(1-a^{\left(i\right)}\right)\right]$$

(4) $$L_{adv}\left(D_{I}\right)=L_{BCE}\left(D_{I}\left(x\right),1\right)+L_{BCE}(D_{I}(G_{1}\left(x\right)),\;0)+L_{BCE}(D_{I}(G_{2}\left(x^{\prime }\right)),\;0)$$

(5) $$L_{adv}\left(G\right)=\frac{1}{2}E_{x\sim pdata(X)}{[\left\|D_{I}(x)-D_{I}(G_{1}\left(x\right))\right\|}_{2}+{\left\|D_{I}(x)-{D_{I}(G}_{2}(x^{\prime }))\right\|}_{2}]$$

Reconstruction loss $L_{rec}$ defines a loss function to minimize the difference between the results created by the generator and the original image (Equation (6)). The difference between all the output images is constructed based on the L1 loss. The minimizing part with x induces the results of $G_{1}\left(x\right)$ and $G_{2}\left(x^{\prime }\right)$ to be similar to *x*.

Typically, the differences between $G_{1}\left(x\right)$ and *x* and between $G_{2}\left(x^{\prime }\right)$ and *x* are generally significant due to the comparison with the original image. However, the comparison between $G_{1}\left(x\right)$ and $G_{2}\left(x^{\prime }\right)$ is crucial as it serves as the basis for judgment in the proposed method, highlighting the differences among the generated images through this comparison. The minimization part between $G_{1}\left(x\right)$ and $G_{2}\left(x^{\prime }\right)$ listed at the end is the most relevant part to the score in the test. The difference in the reconstruction loss characterizes it; thus, it is configured with the lowest ratio.

(6) $$L_{rec}=\frac{1}{3}E_{x\sim pdata(X)}{[\left\|x-G_{1}(x)\right\|}_{1}+{\left\|x-G_{2}(x^{\prime })\right\|}_{1}+{\left\|G_{1}(x)-G_{2}(x^{\prime })\right\|}_{1}]$$

Latent loss $L_{lat}$ is defined similarly to reconstruction loss. Reconstruction loss minimizes the results between image domains, while latent loss minimizes the results between the latent vectors in the feature space. Because the latent vector is the output of the encoder ($E_{1},E_{2},E_{3}$) for each input image and these vectors ($z,z^{\prime },z^{\prime \prime }$) are the result of the compression of the input image, the decoder has the most crucial feature when restoring them. Therefore, it helps each generator produce the same result by minimizing the results of the latent vector. Each part is set based on the L2 loss and appears as Equation (7).

(7) $$L_{lat}=\frac{1}{3}E_{z\sim pdata(Z)}{[\left\|z-z^{\prime }\right\|}_{2}+{\left\|z-z^{\prime \prime }\right\|}_{2}+{\left\|z^{\prime }-z^{\prime \prime }\right\|}_{2}]$$

Total loss, including adversarial loss, reconstruction loss, and latent loss, is expressed as Equation (8). To achieve the best performance, each loss function is multiplied by an appropriate hyperparameter. The values for each hyperparameter are mentioned in Section 4.

(8) $$L_{total}=\lambda_{adv}L_{adv}+\lambda_{rec}L_{rec}+{\lambda_{lat}L}_{lat}$$

## 4. Experiments

### 4.1. Datasets

Among the data provided by AI-Hub [29], a dataset called “Outdoor Crop Disease Diagnostic Image” is used. This dataset is the diseased image data of 10 major open-field crops and provides the JSON with the coordinates for the location of the fruit or leaf (as the metadata). The dataset consists of images taken directly from chili peppers, and we resize them to sizes (128, 128, and 3) for use. Only the data corresponding to chili peppers for the experiment are used among the various classes. However, many data on chili leaves, not only chili fruits, are distributed in the constructed image data. If the datasets are built using only the data on chili pepper berries, only 1351 normal chili pepper images and 975 diseased chili pepper images remain. The network proposed in this paper is insufficient because training should only be conducted with normal chili pepper images. Therefore, we construct a train/test dataset in this paper by augmenting the remaining data. Data augmentation uses only rotation (degrees of 90, 180, and 270) and inversion to expand the number per data by six–seven times for normal chili peppers. The finally constructed training data are 7338 normal chili pepper images. The test data consist of 2000 samples, 1025 normal chili pepper images, and 975 diseased ones.

### 4.2. Detailed Training

The experimental environment in this paper uses the RTX 3090 GPU (Nvidia: Santa Clara, CA, USA) in the operating system environment of Ubuntu 18.04 LTS. It uses the TensorFlow framework and conducts experiments based on version 2.6. The batch size is 128 in all experiments, and the epoch is fixed at 2000. The hyperparameters mentioned in $L_{total}$ and $L_{rec}$ are set to 50 and 1, except $L_{rec}$, to proceed with the training. In addition, the length of the latent vector expressed by compressing the image is set to 100, and the initial learning rate is fixed at 0.0002. The generator and discriminator used in this paper proceed with the same structure as Ganomaly [28] and show a higher performance based on this structure.

### 4.3. Performance Evaluation and Comparisons

**Figure 5:**
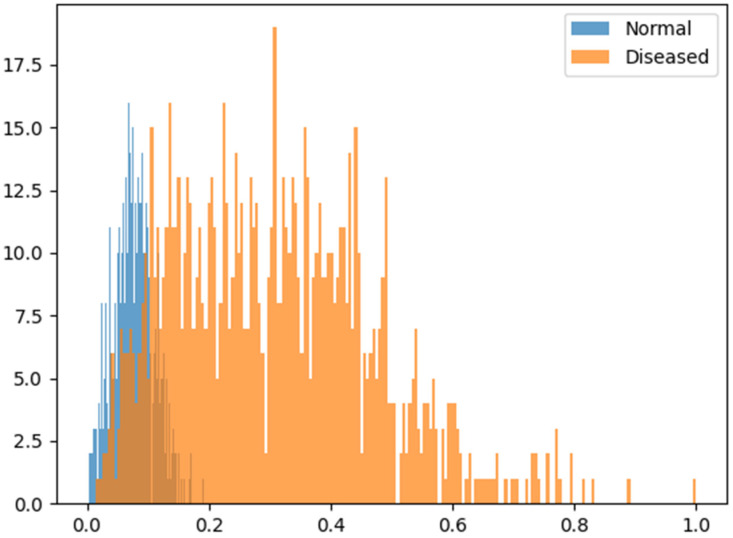
Score distribution.

We introduce the test results using the method proposed in this paper. First, Figure 5 shows the graph of the score for each data point. The graph has a range of [0,1] and consists of 200 bins.

The blue bar represents the score distribution of the normal pepper image, and the orange bar represents the score distribution of the diseased chili pepper image. Overall, each distribution shows a normal distribution with a convex middle region, and the maximum value of the normal chili pepper image does not exceed 0.2 and shows a result close to 0. The expected outcome in analyzing images depicting diseased peppers is that the results should closely approach a value of 1. However, the observed results are uniformly distributed across the interval [0,1], leading to overlapping distributions. Binary classification uses the optimal threshold τ to distinguish between normal and diseased. As mentioned in Equation (8), if it is less than *τ*, it is considered normal; otherwise, it is considered diseased. The optimal threshold τ is calculated to 0.1402.

**Figure 6:**
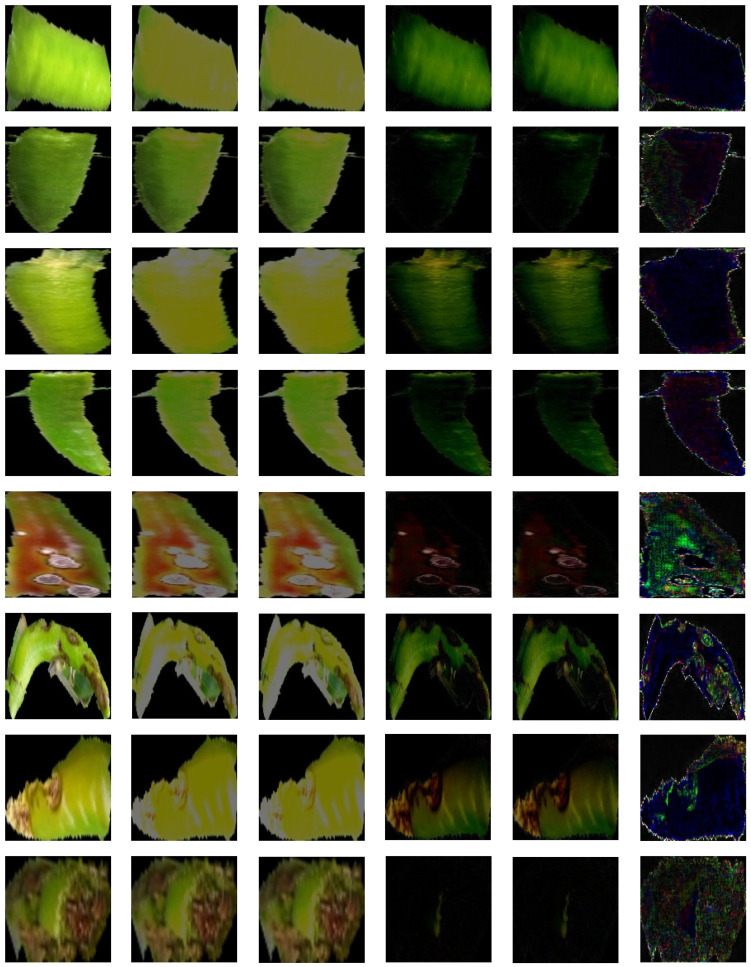
Reconstruction and subtraction results (column 1: input image; columns 2 and 3: reconstruction image, and columns 4–6: subtraction images).

Figure 6 shows the results of the proposed method: column 1 shows the original image and columns 2 and 3 show the results output by the generator. In addition, lines 1 to 4 show the results of the normal chili pepper image, and lines 5 to 8 show the results of the diseased chili pepper image. The difference between the original image and the generated image is blurred or different in color, showing results that cannot be reconstructed in detail. It can be seen that the difference between $G_{1}(x)$ and $G_{2}(x^{\prime })$ is not significant as a visual result.

Overall, the reconstruction results by training the approximate shape and color distribution are shown, and, in particular, the area where light is reflected tends to be enlarged and interpreted. The images in columns 4 to 6 represent the combined difference in images using the results in columns 1 to 3 (column 4: $\left|x-G_{1}(x)\right|$; column 5: $\left|x-G_{2}(x^{\prime })\right|$; and column 6: $\alpha \left|G_{1}\left(x\right)-G_{2}(x^{\prime })\right|$). $\left|x-G_{1}(x)\right|$ represents an original image and a differential image of $G_{1}\left(x\right)$, and $\left|x-G_{2}(x^{\prime })\right|$ represents an original image and a differential image of $G_{2}(x^{\prime })$. Looking at the results, there is no difference, and it can be seen that it is difficult to distinguish between lines 1 to 4 (normal) and 5 to 8 (diseased). Therefore, low performance results will be produced if this is used to calculate the score. The results in column 6 are the results $\alpha \left|G_{1}\left(x\right)-G_{2}(x^{\prime })\right|$ for the differential images of $G_{1}\left(x\right)$ and $G_{2}(x^{\prime })$ used in this paper. Since the difference is very small, the result obtained by multiplying the weight by α is visually shown, and α is set to 20. The results in column 6 show that the overall difference in $\alpha \left|G_{1}\left(x\right)-G_{2}(x^{\prime })\right|$ of the normal chili pepper image is very small, and the blue pixels are slightly distributed. However, the $\alpha \left|G_{1}\left(x\right)-G_{2}(x^{\prime })\right|$ of the diseased chili pepper image shows a noticeable result of the difference. It can be seen that not only are blue pixels distributed but the green pixels appear to be intense in the diseased area. Therefore, it may be seen that the results of the normal and diseased images can be divided into better performances using the differential images of the generators.

Table 2 shows the method proposed in this paper and the performance comparison with baseline studies. Bold text indicates the highest value in each table. The CAE has the same dimension as the dataset as the input by adding the convolutional layers to the autoencoder. It has a network structure based on a U-Net structure with a skip connection structure.

For the experiments, we define a loss function to ensure that the input and output results are identical. Unlike Ganomaly [28] and the proposed method, the reconstruction image of the CAE takes a different form from the original structure. It has the lowest performance because there are many misclassified results. The F1 score shows a low performance of 56.7% (normal) and 68.6% (diseased). Ganomaly [28] is a baseline model for the method proposed in this paper. This research achieved a high performance by calculating the difference between the latent vectors based on features as a score. It shows a significant performance improvement of about 30% compared to the CAE. However, if Ganomaly [28] is calculated as an image-based score, it shows a low F1 score of 21.3% based on normal and 12.2% based on diseased images. Although this differs from the performance of the CAE, it shows that it is difficult to classify based on images in the reconstruction method. However, the method proposed in this paper solved the difficult task. The proposed method shows a higher F1 score than the CAE image reconstruction method, which shows a tremendous performance improvement of 23.7% on normal and 13.7% on diseased images. This is because the score is calculated to optimize the classification based on the threshold. Although it is higher than the performance between features, it is particularly noteworthy that it is solved by an error method between images. The proposed method shows a quantitative evaluation of 90.9% on normal and 89.1% on diseased images with the F1 score. The proposed method shows a high performance of over 95% in precision, especially in recall, for normal and diseased images. It accurately classifies the actual normal chili pepper image, and it can be interpreted that there is very little misclassified content for the results detected as diseased. In terms of normal image precision and diseased image recall, there is a slight performance decline, but, from the perspective of the overall F1 score, it can be seen as a better performance.

The final score is calculated using the results of $G_{1}$ and $G_{2}$. The diseased image is diagnosed using them. The form of a series in which two autoencoder structures are continuously combined results in the corresponding score calculation method showing a great performance improvement. This section also introduces each generator’s combined diagnostic results and the results between the latent vectors. The latent vector is a part that is not noted in this paper, but the analysis is conducted to compare it with previous research. The score calculation method combines three latent vectors and three images, which are analyzed for a total of six types, and the evaluation method is compared using the AUC, AP, and Macro F1. The AUC sets the TPR and FPR to the x- and y-axes, respectively, drawing a Receiver Operating Characteristic (ROC) curve and then obtains the area below it. This area is a numerical value for performance evaluation. The closer it is to 1, the better the model. Unlike the ROC curve drawn with TPR and FPR, AP refers to the area under the graph drawn with recall and precision as the x- and y-axes. AP is mainly used in computer vision: the higher the value, the better. Macro F1 refers to the average of the F1 scores calculated by different positives among several classes. This means the closer it is to 1, the higher the performance.

**Figure 7:**
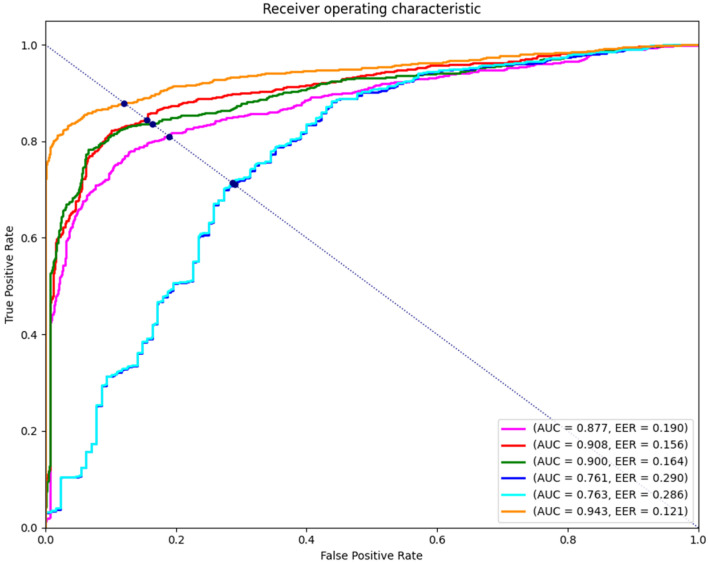
Comparison of ablation study with AUC.

Figure 7 shows the ROC curve for the six score calculation methods. The blue and cyan graphs show the results for $S\left(x,G_{1}(x)\right)$ and $S\left(x,G_{2}(x^{\prime })\right)$. The two results show a very slight difference on the graph. The AUC is 0.761 and 0.763, which is significantly lower than other score calculation methods. The magenta, red, and green show a graph of the scoring method using a latent vector and show results that are significantly higher than scoring based on the image. Magenta is the graph of $S\left(z,z^{\prime }\right)$, red is the graph of $S\left(z,z^{\prime \prime }\right)$, and green is the graph of $S\left(z^{\prime },z^{\prime \prime }\right)$, of which $S\left(z,z^{\prime \prime }\right)$ shows the best performance. The methods $S\left(G_{1}(x),G_{2}(x^{\prime })\right)$ proposed in this paper are the highest performance methods with the highest AUC 94.3% compared to the other methods.

Table 3 shows additional comparison information between the AP and Macro F1, including the abovementioned AUC. $S\left(x,G_{1}(x)\right)$ and $S\left(x,G_{2}(x^{\prime })\right)$ show the lowest performance in the AP and Macro F1. In calculating the score based on the latent vector, $S\left(z,z^{\prime \prime }\right)$ performed the most with an AP of 0.915 and a Macro F1 of 0.861. However, the method proposed in this paper shows the highest performance compared to all of these. The AP is 0.959, and the Macro F1 is 0.900, which is an increase of 0.044 and 0.039 compared to $S\left(z,z^{\prime \prime }\right)$, which showed the best performance in the latent vector. Therefore, $S\left(G_{1}(x),G_{2}(x^{\prime })\right)$ is compared with other score calculation methods and analyzed through several methods, indicating that it is the best method for all areas.

Table 4 presents the quantitative analysis of performance variations based on the presence or absence of different loss functions in the proposed method. Since the model is GAN-based, adversarial loss is a fundamental component. Analyzing the results, it becomes clear that reconstruction loss is necessary to ensure pixel-level reconstruction performance. The model that includes only reconstruction loss (AUC: 0.886, AP: 0.891, and Macro F1: 0.817) demonstrates a high performance, indicating that reconstruction-focused learning has been effectively achieved.

On the other hand, when only latent loss is added without reconstruction loss (AUC: 0.102, AP: 0.310, and Macro F1: 0.512), the model records the lowest performance. This suggests that when latent loss is used alone, the model does not achieve sufficient reconstruction capability within the learned latent space. Moreover, in the proposed method, combining latent loss with reconstruction loss (AUC: 0.943, AP: 0.959, and Macro F1: 0.900) results in a significant performance improvement. This indicates that latent loss contributes to a more structured learning of the latent space, and, when combined with reconstruction loss, it enhances reconstruction performance. In contrast, using only adversarial loss (AUC: 0.286, AP: 0.357, and Macro F1: 0.512) leads to a relatively poor quality of the generated outputs. This shows that focusing solely on the basic GAN training paradigm is insufficient for achieving robust reconstruction, highlighting the limitations in obtaining fine-grained reconstruction performance. While adversarial loss provides the basic framework for generation, it is quantitatively proven that combining it with reconstruction loss and latent loss is essential for achieving an optimal performance.

Therefore, this analysis clearly illustrates the individual roles of each loss function, emphasizing that a balanced use of adversarial loss, reconstruction loss, and latent loss in the proposed method is the key to maximizing performance.

The proposed method is based on the fundamentals of anomaly detection and aims to compare its performance with the latest reconstruction-based research, including State-of-the-Art (SOTA) studies. Table 5 evaluates the results using three key performance metrics: AUC, AP, and Macro F1. The O and X for each method indicate whether GrabCut is used or not.

The analysis reveals that, while the GrabCut [20] technique generally aims to enhance the performance by removing backgrounds, it leads to performance degradation in the cases of DFR [11] and DRAEM [12]. In contrast, V-DAFT [9] and DDAD [13] demonstrate an improved performance with the application of GrabCut [20]. This suggests that research focusing on features is less affected by background noise, whereas studies that do not emphasize this aspect are more susceptible to such noise. The proposed method achieves the highest performance in the AP (95.9%) and Macro F1 (90.0%) metrics. However, in the aspect of AUC (Ours: 94.3%), DDAD [13] shows a marginally higher performance by 0.8%. This implies that, while the threshold setting process for classifying normal and defective patterns in DDAD [13] is somewhat less efficient, the individual sample scores are higher than the proposed method. DRAEM [12] exhibits its best performance without using GrabCut [20], suggesting an advantage in generating anomalous images for segmentation in detecting diseases in peppers. In the case of V-DAFT [9], the influence of the background in the denoising autoencoder process is significant, resulting in a very low AUC of 30.5% when GrabCut [20] is not used. However, using GrabCut [20], which removes the background, increases the AUC to 58.1%.

Although GrabCut [20] has a substantial impact on the proposed method, the serially used generative autoencoder allows for the more accurate calculation of different images. This enables the method to achieve the highest performance compared to other SOTA studies without focusing intensively on features. This indicates that if the proposed method solves the issue of background noise, it has the potential to achieve an excellent performance even when applied to other fields.

## 5. Conclusions

We show a GAN-based training structure in which two generators are connected in series, and the discriminator distinguishes between two fake and one real image. In addition, based on the chili pepper image, the background was removed using GrabCut, and a system for disease diagnosis was shown in the image reconstruction method. The proposed method calculated and evaluated the score using the error between the reconstructed images rather than the latent vector. It shows a very high-performance improvement from the perspective of the image, unlike previous research. This approach has the advantage of allowing the detection of diseases without requiring expert knowledge, making it easy to identify the presence or absence of diseases. However, since the dataset used has a limited color or type of pepper, it is necessary to expand and conduct research. In addition, automatically extracting the bounding box coordinates required by GrabCut will attract attention in the smart farm field if it is linked to automatically detecting the location of peppers in connection with object detection in the future. Furthermore, the proposed method achieved the best performance in two out of the three metrics, indicating that utilizing the diffusion model for generation may provide a better reconstruction performance compared to the GAN. Therefore, by modifying the generation method in the proposed approach to use the diffusion model, an even higher performance could be expected.

## Figures and Tables

**Table 1:** Model architecture and detailed information.

Module	Kernel Size	Stride	Padding	Normalization	Activation Function	Feature Map
$E_{1},\;E_{2},\;E_{3}$,$\;D_{I}$	4 × 4	2	same	BN	Leaky-ReLU	(64, 64, 64)
	4 × 4	2	same	BN	Leaky-ReLU	(32, 32, 128)
	4 × 4	2	same	BN	Leaky-ReLU	(16, 16, 256)
	4 × 4	2	same	BN	Leaky-ReLU	(8, 8, 512)
	4 × 4	2	same	BN	Leaky-ReLU	(4, 4, 1024)
	4 × 4	1	valid	BN	Leaky-ReLU	(1, 1, 100)
$D_{1},\;D_{2}$	4 × 4	1	valid	BN	ReLU	(4, 4, 512)
	4 × 4	2	same	BN	ReLU	(8, 8, 256)
	4 × 4	2	same	BN	ReLU	(16, 16, 128)
	4 × 4	2	same	BN	ReLU	(32, 32, 64)
	4 × 4	2	same	BN	ReLU	(64, 64 32)
	4 × 4	2	same	BN	Tanh	(128, 128, 3)

**Table 2:** Comparisons with baseline works.

Method		Precision	Recall	F1 Score
CAE	Normal	0.727	0.464	0.567
	Diseased	0.592	0.816	0.686
Ganomaly [28] (based on features)	Normal	**0.875**	0.895	0.885
	Diseased	0.887	0.866	0.876
Ganomaly [28] (based on images)	Normal	0.836	0.562	0.672
	Diseased	0.658	**0.884**	0.754
Proposed method	Normal	0.859	**0.966**	**0.909**
	Diseased	**0.959**	0.833	**0.891**

**Table 3:** Comparison of the results of the score calculation methods.

Method	$S\left(z,z^{\prime }\right)$	$S\left(z,z^{\prime \prime }\right)$	$S\left(z^{\prime },z^{\prime \prime }\right)$	$S\left(x,G_{1}(x)\right)$	$S\left(x,G_{2}(x^{\prime })\right)$	$S\left(G_{1}(x),G_{2}(x^{\prime })\right)$
AUC	0.877	0.908	0.900	0.761	0.763	**0.943**
AP	0.879	0.915	0.910	0.699	0.701	**0.959**
Macro F1	0.826	0.861	0.859	0.712	0.713	**0.900**

**Table 4:** Accuracy comparison based on the use of loss functions.

$L_{adv}$	O	O	O	O
$L_{rec}$	X	O	X	O
$L_{lat}$	X	X	O	O
AUC	0.286	0.886	0.102	**0.943**
AP	0.357	0.891	0.310	**0.959**
Macro F1	0.512	0.817	0.512	**0.900**

**Table 5:** Comparative analysis of reconstruction-based anomaly detection methods.

Method	V-DAFT [9]		DFR [11]		DRAEM [12]		DDAD [13]		Ours	
GrabCut	X	O	X	O	X	O	X	O	X	O
AUC	0.305	0.581	0.897	0.853	0.911	0.820	0.906	**0.951**	0.304	0.943
AP	0.224	0.538	0.855	0.847	0.883	0.861	0.772	0.933	0.231	**0.959**
Macro F1	0.242	0.586	0.848	0.771	0.875	0.784	0.843	0.895	0.417	**0.900**

## Data Availability

Data are contained within the article.
